# ANCA: A Web Server for Amino Acid Networks Construction and Analysis

**DOI:** 10.3389/fmolb.2020.582702

**Published:** 2020-11-19

**Authors:** Wenying Yan, Chunjiang Yu, Jiajia Chen, Jianhong Zhou, Bairong Shen

**Affiliations:** ^1^Center for Systems Biology, School of Biology and Basic Medical Sciences, Soochow University, Suzhou, China; ^2^School of Biotechnology, Suzhou Industrial Park Institute of Services Outsourcing, Suzhou, China; ^3^School of Chemistry, Biology and Materials Engineering, Suzhou University of Science and Technology, Suzhou, China; ^4^Public Library of Science, San Francisco, CA, United States; ^5^Institutes for Systems Genetics, West China Hospital, Sichuan University, Chengdu, China

**Keywords:** Amino acids network, ANCA portal, network analysis, protein structure, allosteric regulation, functional residues

## Abstract

Amino acid network (AAN) models empower us to gain insights into protein structures and functions by describing a protein 3D structure as a graph, where nodes represent residues and edges as amino acid interactions. Here, we present the ANCA, an interactive Web server for Amino Acids Network Construction and Analysis based on a single structure or a set of structures from the Protein Data Bank. The main purpose of ANCA is to provide a portal for three types of an environment-dependent residue contact energy (ERCE)-based network model, including amino acid contact energy network (AACEN), node-weighted amino acid contact energy network (NACEN), and edge-weighted amino acid contact energy network (EACEN). For comparison, the C-alpha distance-based network model is also included, which can be extended to protein–DNA/RNA complexes. Then, the analyses of different types of AANs were performed and compared from node, edge, and network levels. The network and corresponding structure can be visualized directly in the browser. The ANCA enables researchers to investigate diverse concerns in the framework of AAN, such as the interpretation of allosteric regulation and functional residues. The ANCA portal, together with an extensive help, is available at http://sysbio.suda.edu.cn/anca/.

## Introduction

With the increasing number of high-resolution 3D structures of biomolecules, including proteins, protein–DNA complexes, and protein–RNA complexes, the development of rapid and efficient methods to perform large-scale analysis for them is needed. A variety of structure-based computational tools and methods is developed to satisfy the new challenges (Romero-Rivera et al., 2016; Liu et al., 2019; Sequeiros-Borja et al., 2020), such as consensus-based, machine learning-based, molecular dynamics (MD) simulation-based, quantum-mechanic simulation-based methods, and so on. The network concepts and methods have been widely used in numerous problems in different fields of biological science including the study of protein structures and functions (Hu et al., 2017). Amino acid network (AAN) models, which are undirected networks consisting of amino acid residues and their interactions, have opened numerous opportunities to reveal new insights in understanding the function of biomolecules from large-scale 3D structure data. Compared with traditional structure-based methods, studying a biomolecule from a network perspective not only gives a systems-level understanding of the biomolecule structure through topological information and global connectivity (Yan et al., 2014; Zhou et al., 2014) but also provides an efficient way for characterization of each individual amino acid within the complex interaction network, such as protein–protein interfaces (Di Paola et al., 2015), catalytic residues (Zhou et al., 2016), and allosteric regulation (Di Paola and Giuliani, 2015; Yan et al., 2018).

Nowadays, several Web tools that construct different types of AANs have facilitated progress in this area of research. RING2.0 constructs an AAN based on the physicochemical interactions between the residues, which include covalent and non-covalent interactions (Piovesan et al., 2016). Protein contact atlas focuses on the non-covalent interactions within structures and shows them at different scales ranging from atomic level to the entire macromolecule level (Kayikci et al., 2018). webPSN investigates structural communication in macromolecules by constructing static and dynamic AANs (Felline et al., 2020). Furthermore, AAN-based Web servers such as MDN (Ribeiro and Ortiz, 2015), NAPS (Chakrabarty and Parekh, 2016), and RIP-MD (Contreras-Riquelme et al., 2018) provide tools for quantifying protein dynamic based on MD simulation trajectories. More tools and Web servers for network can be found in a recent review (Liang et al., 2020). However, many of the AAN models only considered amino acid interactions on a geometric level but not on the chemical properties of the proteins. An alternative strategy for the simulation of the interactions is using the energy between residues. We proposed an amino acid contact energy network (AACEN) based on a coarse-grained contact energy called environment-dependent residue contact energy (ERCE; Yan et al., 2014), which takes into account the type of secondary structure for each residue and is more efficient and easier for characterizing the energy between residues (Zhang and Kim, 2000; Shen and Vihinen, 2003). Moreover, another inadequacy of most AAN models is the disregard for heterogeneity of residues and treating all nodes as the same in the network. To address this, we improved our AACEN model by assigning residue properties as node weights and named it as node-weighted amino acid contact energy network (NACEN; Yan et al., 2018).

**FIGURE 1:**
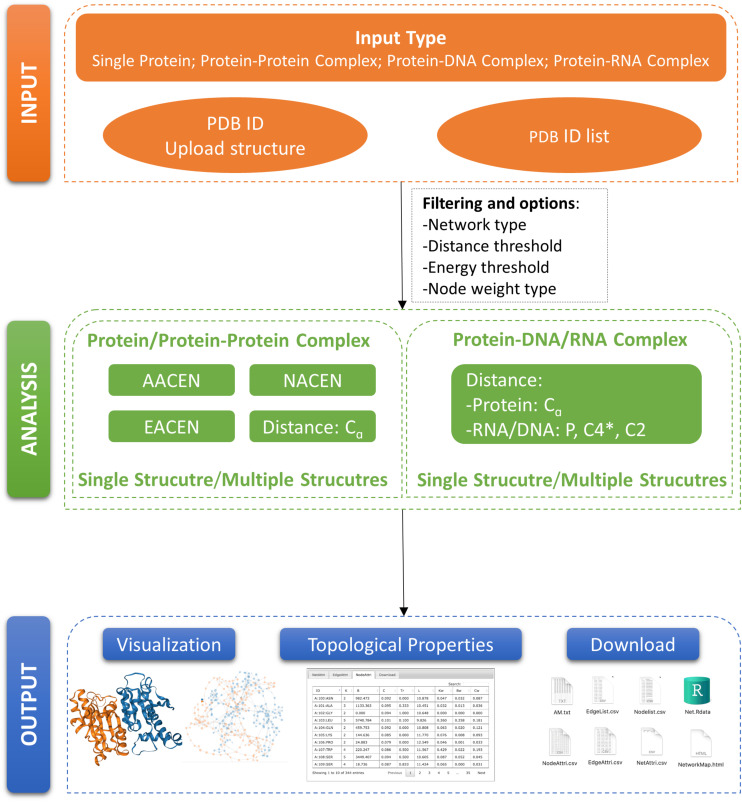
Organization of ANCA (Amino Acids Network Construction and Analysis). A schematic view of ANCA input, analysis, and output is provided here.

In this paper, we developed a Web server called ANCA (Amino Acids Network Construction and Analysis) for construction and analysis of our previously proposed ERCE-based network models AACEN and NACEN. To refine our ERCE-based models, we also added the edge-weighted amino acid contact energy network (EACEN) model in our Web server using the ERCE as link weights. Moreover, a C-alpha distance-based network (C-alpha) model was also included in our ANCA for two purposes. Firstly, the C-alpha model can be used as the comparison network for our ERCE-based models for proteins or protein complexes. Secondly, ANCA also provides the construction and analysis for single and multiple protein–DNA/RNA complexes based on the C-alpha model. The organization of our portal ANCA was shown in Figure 1.

## Methods and Implementation

The ANCA Web server is comprised of two core modules entitled “single structure” and “multiple structures.” The single-structure module provides the construction and analysis for one structure with any one of the AAN models at a time, while in the multiple-structures module, the structures can be analyzed in batches using any of the four types of AAN models. The former module is more suitable for carrying out a detailed analysis for one structure (either a PDB code or a PDB file). The latter module can be used for comparison analysis of different structures. Both modules support four types of AAN construction, analysis, and visualization. Moreover, ANCA can provide the option of distance-based AAN construction for protein–DNA/RNA complexes.

### Amino Acid Network

#### Amino Acid Contact Energy Network

As defined in our previous studies (Yan et al., 2014; Zhou et al., 2014), an amino acid residue in the protein or protein complex is denoted as a node and a link is set to two nodes if the ERCE (Zhang and Kim, 2000; Shen and Vihinen, 2003) between them is less than 0. ERCE is an improvement of Miyazawa–Jernigan’s model by an extension of residue alphabet from 20 to 60, which considers the 20 amino acids in three secondary structural states. ERCE *e*_*ij*_ between residues *i* and *j* was defined as in our previous studies (Yan et al., 2014), and then according to the *e*_*ij*_, the element in the adjacent matrix AM of AACEN was set to 1 if *e*_*ij*_ was less than 0, otherwise the element was set to 0 (Yan et al., 2018).

#### Node-Weighted Amino Acid Contact Energy Network

Based on AACEN, we have developed a NACEN module to characterize and predict functional residues. In this network representation, links between residues were defined the same as in AACEN, and the properties of residues, including relative solvent accessibility (SAS), mass, hydrophobicity, polarity, or user-self defined node weights (Yan et al., 2018).

#### Edge-Weighted Amino Acid Contact Energy Network

In EACEN, the links between residues were weighted by ERCE and the adjacent matrix AM of EACEN was defined as:

(1) $${\text{AM}}_{i\,j}=\left\{ \begin{array}{c} 0,e_{i\,j}\geq 0\\ w_{i\,j},e_{i\,j}<0 \end{array}\right.$$

Where *w*_*ij*_ is the normalization of the contact energy *e*_*ij*_ between *i* and *j*:

(2) $${\text{w}}_{i\,j}=\left\{ \begin{array}{c} 0.0001,i\,f\,\left|e_{i\,j}\right|={\left|e_{i\,j}\right|}_{m\,i\,n}\\ \frac{\left|e_{i\,j}\right|-{\left|e_{i\,j}\right|}_{m\,i\,n}}{{\left|e_{i\,j}\right|}_{m\,a\,x}-{\left|e_{i\,j}\right|}_{m\,i\,n}},i\,f\,\left|e_{i\,j}\right|\neq {\left|e_{i\,j}\right|}_{m\,i\,n} \end{array}\right.$$

#### C-Alpha Distance-Based Network

ANCA can construct the network for protein–protein, protein–DNA, or protein–RNA complex based on the distance between represented atoms. For a protein–protein complex, the link between two residue nodes in the network was established if the distance between C-alphas of the residues is lower than a threshold (Di Paola et al., 2013). For a protein–DNA complex or protein–RNA complex, we use one node to represent one amino acid, and three nodes of P, C4^∗^(sugar group), and C2 (base group) atoms to represent each nucleotide of the DNA or RNA (Delarue and Sanejouand, 2002).

As mentioned above, our portal provides the above four AAN models, including two unweighted AANs (C-alpha and AACEN) and two weighted AANs (NACEN and EACEN). The C-alpha model can be used not only for protein and protein–protein complex but also for protein–DNA/RNA complex. But since the network is constructed just based on the distance between C-alpha atoms, it is a relatively coarse model. AACEN is an ERCE-based network model that can be used just for protein or protein complex but provide more detailed information by considering the local environment of the residues (Zhang and Kim, 2000), and it has been used to compare protein structures and evolution (Yan et al., 2014, 2016). NACEN and EACEN are also ERCE based. The difference is the former one also employs the characters of residues as node weights, so it is more suitable to explore residue function (Yan et al., 2018), while the latter one assigns the ERCE between residues as the link weights that provide more detailed information on the links between residues than the unweighted model, so it can be helpful for studying the communication between residues, such as allosteric regulation.

### Analysis and Visualization of the Amino Acid Network

ANCA can be used for the network analysis of proteins, protein–protein complexes, and protein–DNA/RNA complexes from the node level, edge level, and network level. The detailed definition of the parameters was listed in http://sysbio.suda.edu.cn/anca/.

At node level, the topological parameters of nodes are calculated, including degree, betweenness, closeness, transitivity, and average shortest path length (*L*_*net*_). Moreover, for NACEN, we also calculated the weighted degree, betweenness, and closeness centralities based on the node weights. Their definitions were in our previous work (Yan et al., 2018). At the edge level, edge betweenness centrality is calculated for each edge to evaluate the importance of the edge. Moreover, the long-range link, which is related to protein secondary structure density and residue evolution rate (Yan et al., 2014), is also labeled. At the network level, the node number (n), edge number, *L*_*net*_, density, and diameter of the network are calculated.

The ANCA provides two types of visualization for protein molecule 3D structure and AAN. ANCA uses NGL Viewer (Rose and Hildebrand, 2015) to display the protein molecule 3D structure using the NGL JavaScript library. The visualization for the AAN is implemented using R package networkD3 (Allaire et al., 2017). In the AAN view, the lines represent the edges and the dots represent the amino acid. When the mouse pointer hovers over the dot, the amino acid name will be shown beside the dot. Users can use the mouse to manipulate the graph, such as scroll mouse wheel to zoom in or out of the graph, move the mouse by pressing the left button to rotate or drag the graph, and so on. The color of the protein molecule 3D structure and the AAN can consist with the chain name.

### Implementation

The ANCA portal can be accessed by modern popular Web browsers, including Chrome, Internet Explorer, Safari, and Firefox, without installing any specialized software or browser plug-ins. The Apache^1^ was used as the Web server, which is a secure, efficient, and extensible open-source HTTP server. The application was realized using three-tiered architecture. In the view tier, the front-end program was developed using PHP^2^, the user interface interaction was realized using jQuery^3^, and the advanced interaction control DataTable^4^ was adopted to represent the result data. In the controller tier, we used C# and.NET Framework 4.0^5^ to implement the logic process program, and the R program was used to construct and analyze the AANs. In the model tier, MySQL^6^ was used to store execution-related information.

## Workflow of ANCA

### Step 1: Module Selection

**FIGURE 2:**
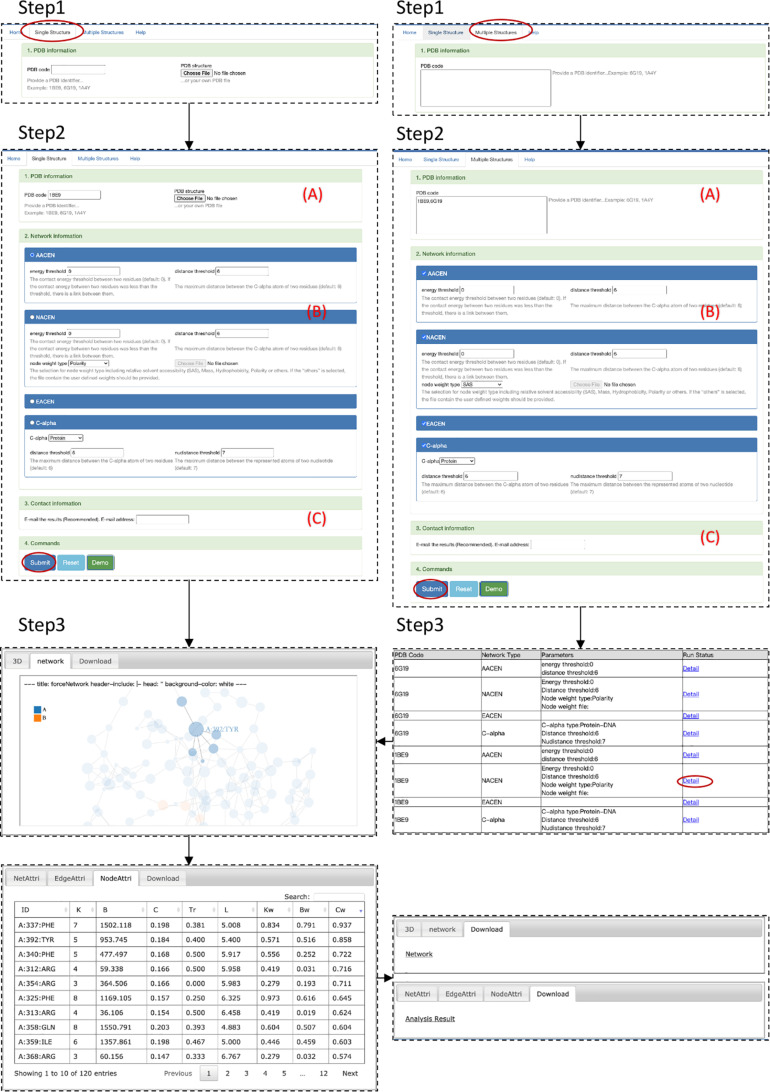
Step-by-step workflow of ANCA.

The step-by-step workflow of ANCA is shown in Figure 2. The first step is module selection for single structure or multiple structures (step 1 in Figure 2). Both modules support the four types of AAN construction and analysis including AACEN, NACEN, EACEN, and C_*upalpha*_ distance-based AAN. In the single-structure module, one of the AAN is constructed. The results page shows the topological properties of the AAN and the visualization of structure and network. While in multiple-structures module, any type from the four AAN models can be constructed and analyzed for each structure, and the results page will demonstrate summary information for all the AANs.

### Step 2: Input Data Upload and Parameter Setting

This step contains three procedures (Figure 2). First, PDB ID or file in PDB format of the structure should be filled in or uploaded (A in Figure 2). The input file should have a.pdb extension. The second procedure (B in Figure 2) is the selection of AAN type, i.e., AACEN, NACEN, EACEN, and C-alpha distance-based AAN. Then, the parameters of the corresponding AAN type should be specified. For AACEN and NACEN, the threshold of energy and distance between residues should be set. Besides these two parameters, users should also specify the node weights of residues either by selecting the default properties of residues, including SAS, mass, hydrophobicity, and polarity or by uploading the file (.txt) that contains user-self defined property. Lastly, the e-mail address can be optionally provided (C in Figure 2) that will be used to receive the results page link from ANCA portal. More detailed description is available at http://sysbio.suda.edu.cn/anca/

### Step 3: Output Description

The output of the ANCA is composed of three parts: visualization, network information, and network topological properties. For visualization, the protein structure and corresponding network are shown in the results page of the Web server, and both of them are colored by the chain of structure. For network information, the results page provides files with adjacent matrix, edge list, and node list of the network. For network topological properties, the ANCA provides the parameters from the node level, edge level, and network level, which have been shown in the Analysis and Visualization of the Amino Acid Network section.

## Case Study

To evaluate the performance of ANCA, we carried out case studies for the single-structure module and multiple-structures module separately, as follows:

### Case 1 Single-Structure Module: Node-Weighted Amino Acid Contact Energy Network for Human PDZ Domain

**FIGURE 3:**
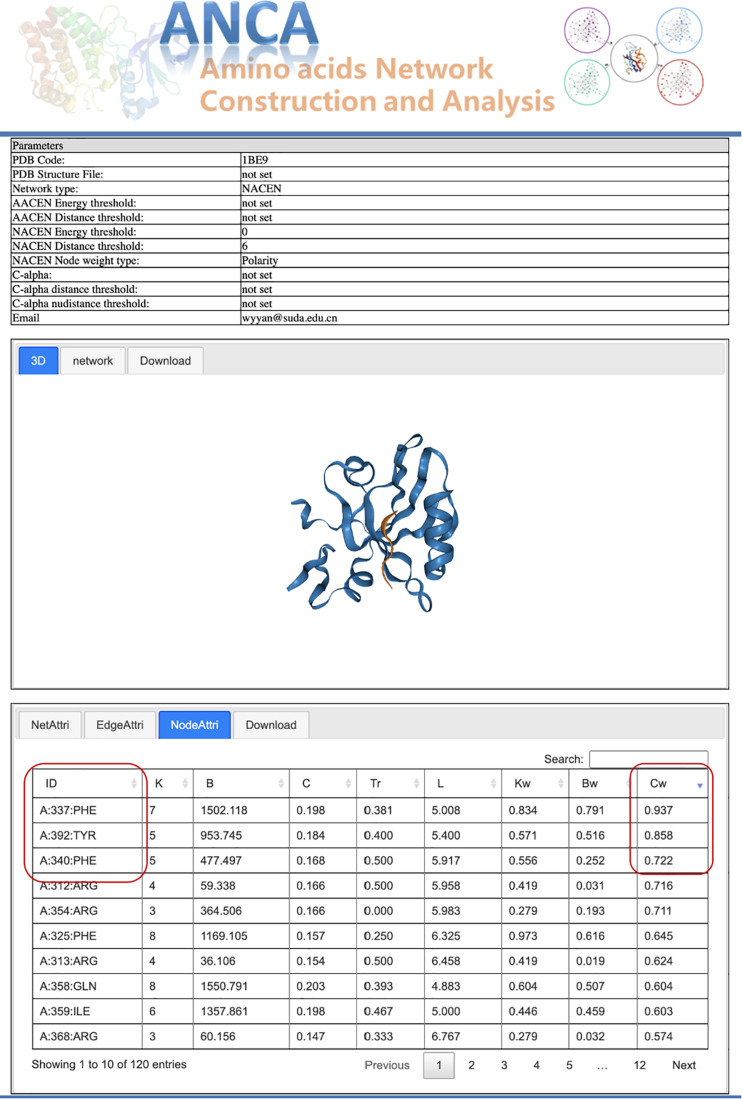
Results page for the case study.

Postsynaptic density-95/Discs large/Zonula occludens 1 (PDZ) protein domain family is a protein–protein interaction module, which is involved in dynamic regulation of signaling pathways and scaffolding and has emerged as a paradigmatic model system for intra-domain allostery (Reynolds et al., 2011; McLaughlin<suffix>Jr.</suffix>, Poelwijk et al., 2012). Here we tried to use our portal ANCA to investigate the allosteric residues of the third PDZ domain of PSD-95 (PDB 1BE9) by the NACEN model. As shown in Figure 2, we chose the single-structure module and NACEN network type with default threshold and selected polarity as node weights. Then, the network and protein structure can be visualized and the topological parameters of the network were listed. At last, the residues were ordered by the weighted closeness centrality (*C*_*w*_), and the results showed that the top 3 residues were PHE337, TYR392, and PHE340 as shown in Figure 3. Among the top 3 residues, two of them, TYR392 and PHE340, have been validated as allosteric residues by double-mutant cycle analysis (Gerek and Ozkan, 2011).

### Case 2 Multiple-Structures Module: All Network Types for Multiple Structures

At this part, we constructed and analyzed all the four types of network, including AACEN, NACEN, EACEN, and C-alpha network for two structures, PDZ3 in case 1 and protein–RNA complex MDA5 double-stranded RNA Filament (PDB 6G19) with default parameters at one time. Then, ANCA portal gave the results page with summary information for each network and the link of the network in the column “Run Status.” The links point to the page containing detailed information for each network as described in case 1.

## Conclusion

ANCA is a comprehensive portal for the construction and analysis of network representations of protein and protein–protein/DNA/RNA complexes to explore and understand the macromolecules at different levels of organization. It can help in the management of heterogeneous information sources, such as structural, sequence, physicochemical, and dynamical information of residues. Another advantage of our portal is that it also allows scientists to address diverse questions by choosing different network models. For example, NACEN is more suitable to identify the functional residues in the structures, while EACEN can capture the intramolecular information flow to help in understanding the allosteric regulation.

## Data Availability Statement

Publicly available datasets were analyzed in this study. This data can be found here: http://sysbio.suda.edu.cn/anca/.

## Author Contributions

WY and BS conceived and designed the Web server. CY performed the server front-end and back-end. WY carried out the case studies. JZ and JC contributed to the Web server testing. WY, CY, and JC wrote the manuscript. JC polished the language and gave many constructive suggestions. BS critically reviewed and edited the manuscript. All authors contributed to the article and approved the submitted version.

## Conflict of Interest

The authors declare that the research was conducted in the absence of any commercial or financial relationships that could be construed as a potential conflict of interest.

## Footnotes

1 http://www.apache.org/

2 https://www.php.net/

3 https://jquery.com

4 https://www.datatables.net/

5 https://docs.microsoft.com/en-us/dotnet/csharp/

6 https://www.mysql.com/
